# Vaccination in the Army

**Published:** 1898-10

**Authors:** W. B. Clarke

**Affiliations:** Indianapolis, Ind.


### VACCINATION IN THE ARMY.

TO THE EDITOR—SIR:—In Surgeon Senn's interesting letter regarding sickness in the army, published by you, is the following notable point: "Almost the first men sent him (General Miles) were from Chickamauga. They were run down and half sick when they arrived. They were in no condition to stand the Southern climate. General Miles noted their weakness."

One reason for this is not hard to find if we look in the right place. These men were the physical flower of the whole country, selected, after rigid inspections, for their high health and superior physical development and vigor, and they were sent to a camping ground that has no superior in the country for large bodies. In opposition to all the rational sanitary laws of our being, they were, with a superstitious imbecility and sen(n)ility worthy of the dark ages, immediately ordered to undergo a process of septic poisoning, so that "the whole current of their blood was touched corruptibly." The very citadel of their life-force was attacked at its weakest point, and their wall of defense broken down, and they became an easy prey to disease. Shakespeare says: "We sicken to prevent sickness when we purge," and when we vaccinate we do the same—only in the latter case we are attacking only a shadow, a possibility of an evil. Our gallant soldiers had real foes enough to meet without compelling them to fight imaginary ones. Whole regiments at a time were thus disabled by this dreadful ogre, and quite a number of men thus lost an arm, though never in battle. Many have not yet recovered from its effects, and many never will. Other countries have learned this terrible lesson, and we should profit by their experience. In Sweden, Norway, Belgium, Holland, and Switzerland no soldier is now obliged to be vaccinated. The vaunted protective power of vaccination has been proved to be a myth, and it has rather proved as Dr. Oidtmann, staff surgeon of the French army at the siege of Paris, in 1871, says: "Vaccination tended rather to extend small-pox in the army than to protect from it."

The bacteriologists now tell us that the festive mosquito transmits malaria and leprosy through his exquisitely attuned trombonic proboscis; that the rapidly-scuttling, turtle-like bedbug carries consumption in his odoriferous bite, and that the busy fly often blood-poisons us to death in a day or two, so it is not in order for them to deny that the meddling vaccinator's lance, loaded with pus from a foul sore on an animal, is the innocent and beneficent instrument its imitative users would have us believe. We learn by our mistakes, and vaccination is one of them. Why be so slow and timid in acknowledging this, and persist in perpetuating the error?

W. B. Clarke, M. D.

Indianapolis, Ind., September 4th.

From the *Indianapolis Sentinel*, September 5th, 1898.

